# Radioiodination of BODIPY and its application to a nuclear and optical dual functional labeling agent for proteins and peptides

**DOI:** 10.1038/s41598-017-03419-z

**Published:** 2017-06-13

**Authors:** Masahiro Ono, Hiroyuki Watanabe, Yuki Ikehata, Ning Ding, Masashi Yoshimura, Kohei Sano, Hideo Saji

**Affiliations:** Department of Patho-Functional Bioanalysis, Graduate School of Pharmaceutical Sciences, Kyoto University, 46-29 Yoshida Shimoadachi-cho, Sakyo-ku, Kyoto, 606-8501 Japan

## Abstract

In molecular imaging research, the development of multimodal imaging probes has recently attracted much attention. In the present study, we prepared radioiodinated BODIPY and applied it as a nuclear and optical dual functional labeling agent for proteins and peptides. We designed and synthesized [^125^I]BODIPY with a *N*-hydroxysuccinimide (NHS) ester, and evaluated its utility as a nuclear and fluorescent dual labeling agent for proteins and peptides. In the radioiodination reaction of BODIPY-NHS with [^125^I]NaI, [^125^I]BODIPY-NHS was obtained at a 48% radiochemical yield. When we carried out the conjugation reaction of [^125^I]BODIPY-NHS with bovine serum albumin (BSA) and RGD (Arg-Gly-Asp) peptide as a model protein and peptide, respectively, [^125^I]BODIPY-BSA and [^125^I]BODIPY-RGD peptide were successfully prepared at 98 and 82% radiochemical yields, respectively. Furthermore, we prepared [^123^I]BODIPY-trastuzumab by this conjugation reaction and successfully applied it to single photon emission computed tomography (SPECT) imaging studies using tumor-bearing mice, suggesting that radioiodinated BODIPY-NHS serves as a dual functional labeling agent for proteins and peptides. Since iodine has various radioisotopes that can be used for SPECT and positron emission tomography (PET) imaging, biological research, and radiotherapy, the radioiodinated BODIPY may be extensively applicable from basic to clinical research.

## Introduction

**Figure 1:**
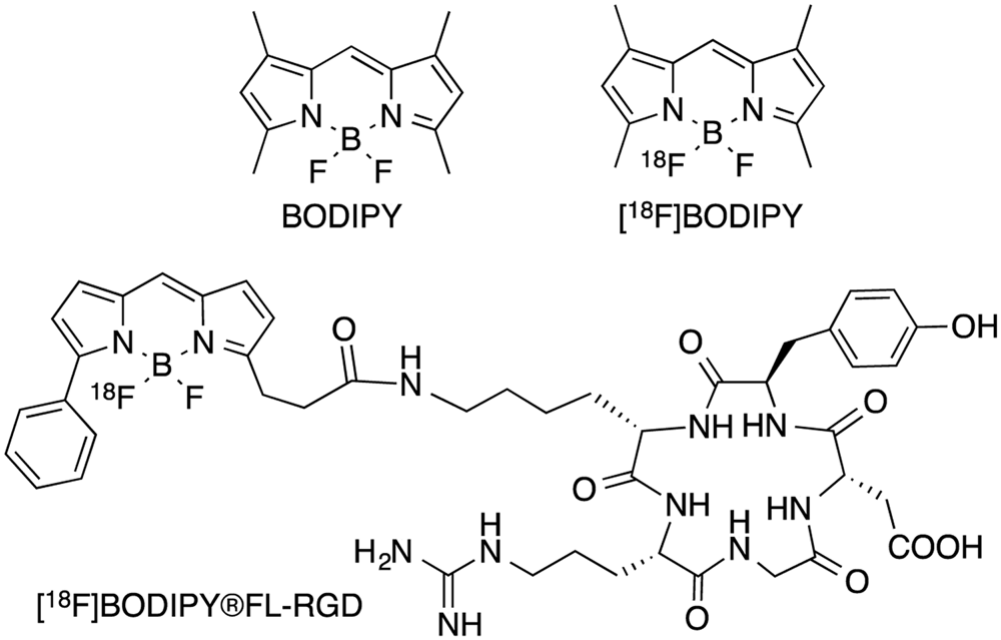
Chemical structures of BODIPY, [^18^F]BODIPY, and [^18^F]BODIPY®FL-RGD.

The *in vivo* quantitative detection of the biodistribution of biologically active proteins and peptides plays an important role in a wide range of research in life science. In such research, the *in vivo* pharmacokinetics of proteins and peptides labeled with radionuclides or fluorescent dye are determined using a nuclear or optical imaging technique. Nuclear imaging techniques including positron emission tomography (PET) and single photon emission computed tomography (SPECT) constitute some of the most common clinical imaging modalities because they can offer a variety of important information on the *in vivo* biodistribution of radiolabeled, biologically active proteins and peptides^1, 2^. In contrast, optical imaging with fluorescent probes has become a versatile modality that has been used for clinical intraoperative tumor detection and *in vivo* imaging studies using small animals^3^. Since both nuclear and optical imaging methods have distinctive characteristics appropriate for clinical application, a dual modality imaging system that combines these two imaging techniques may noninvasively provide complementary information for the diagnosis of various diseases. Many dual imaging probes that can be applied to such nuclear and optical dual modality imaging have been reported^4–11^. Among them, Li *et al*. recently investigated the radiofluorination of BODIPY, which is one of the well-known fluorescent dyes (Fig. 1)^12^. They reported that one of two fluorine atoms in the boron center of BODIPY can be used for radiofluorination, and applied [^18^F]BODIPY as a dual modality imaging agent for the first time (Fig. 1). [^18^F]BODIPY is an attractive dual imaging agent because it can be integrated with positron emission tomography and the optical properties of the imaging agent in the same molecule. Several dual imaging probes with [^18^F]BODIPY including [^18^F]BODIPY®FL-RGD (Fig. 1) have been reported^13–15^. However, the relatively short half-life of ^18^F (t_1/2_ = 110 min) makes the use of [^18^F]BODIPY limited. In particular, it may not be appropriate to determine the *in vivo* pharmacokinetics of proteins such as antibodies using this approach with [^18^F]BODIPY conjugates because, in general, the half-life of such proteins in the blood does not match that of ^18^F. Therefore, to determine the pharmacokinetics of proteins conjugated with [^18^F]BODIPY, it is necessary to develop new agents that can label the proteins with other radionuclides with a longer half-life than ^18^F.

In numerous studies regarding the chemistry of BODIPY^16^, we found that it is possible to introduce iodine into pyrrole rings of the BODIPY scaffold^17, 18^. Since iodine has several radionuclides, including ^123^I (t_1/2_ = 13.2 h, γ), ^124^I (t_1/2_ = 4.18 d, β^+^), ^125^I (t_1/2_ = 59.4 d, Auger e^−^), and ^131^I (t_1/2_ = 8.02 d, β^−^), it can be applied to broad research from basic research to diagnostic imaging and clinical radiotherapy in consideration of their half-life and characteristics of radiation^19^. Therefore, we considered that radioiodinated BODIPY can be used to develop a new method for the dual functional labeling of biologically active peptides and proteins.

In the present study, we synthesized radioiodinated BODIPY ([^123/125^I]BODIPY) and applied it as a nuclear and optical dual functional labeling agent. We selected bovine serum albumin (BSA), RGD peptide, and trastuzumab as a model protein, peptide, and biologically active protein, respectively, to validate the basic concept for the development of new dual functional probes based on the radioiodinated BODIPY, and evaluated the feasibility of using [^123/125^I]BODIPY as a dual functional labeling agent for peptides and proteins.

## Results and Discussion

### Synthesis and characterization of ^125^I-labeled BODIPY ([^125^I]2)

**Figure 2:**
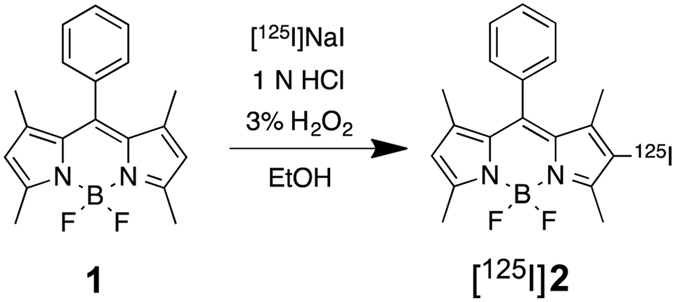
Radioiodination reaction of BODIPY.

**Figure 3:**
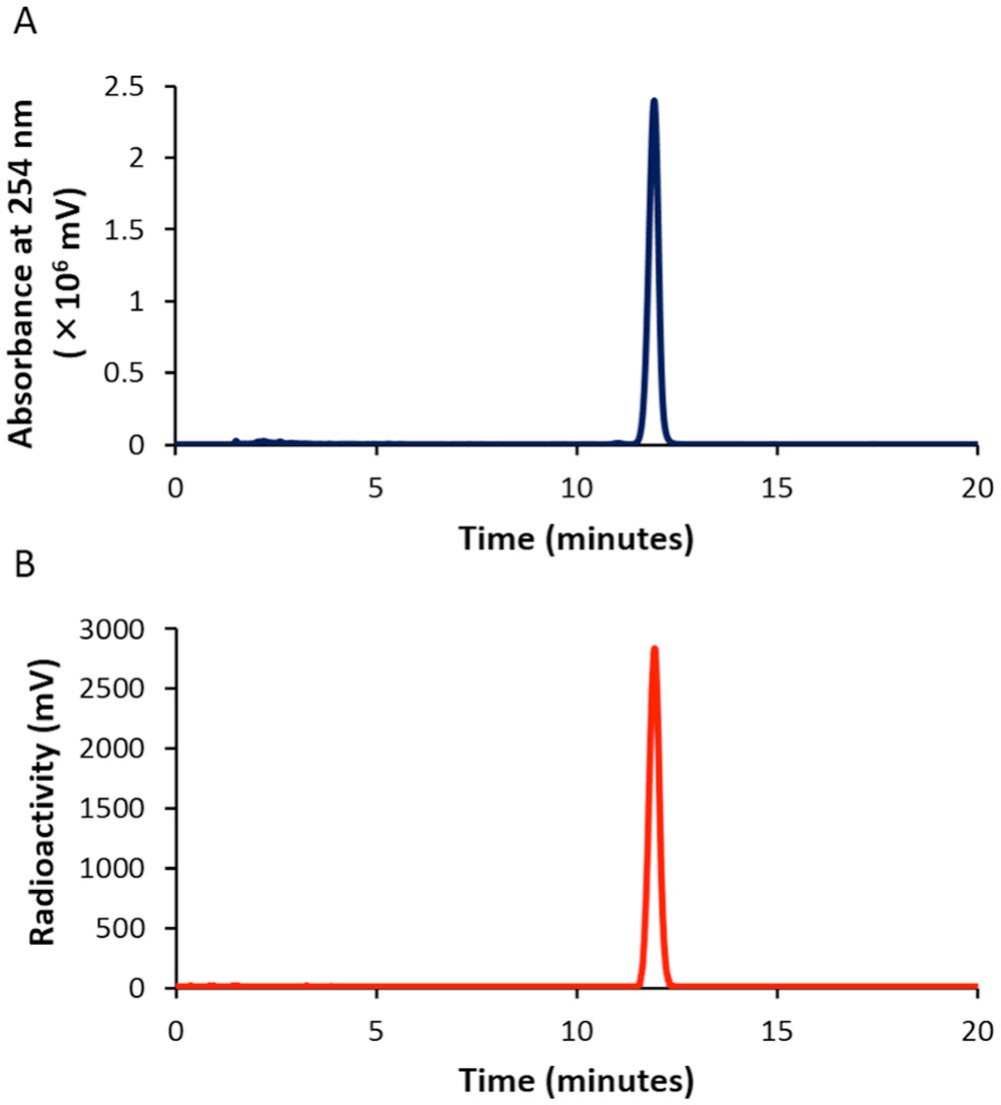
HPLC profiles of **2** (absorbance at 254 nm, **A**) and [^125^I]**2** (radioactivity, **B**).

We carried out a radioiodination reaction according to the synthetic route shown in Fig. 2. The BODIPY scaffold (**1**) was radioiodinated with [^125^I]NaI in the presence of hydrogen peroxide as the oxidant. The radiochemical identity of ^125^I-labeled BODIPY ([^125^I]**2**) was verified by the HPLC profile of nonradioactive iodo-BODIPY (**2**), which was synthesized according to a method reported previously (Fig. 3)^17^. We efficiently prepared [^125^I]**2** at a radiochemical yield of 75.1% with a radiochemical purity of >99% after purification by HPLC. This efficiency of radiolabeling was similar to that obtained with the conventional radioiodination including electrophilic reactions with activated aromatic groups, and iodo-demetalation of aryls and alkyenes, using organotin or silicon precursors^19, 20^. The new radioiodination reaction of BODIPY may be applicable for a variety of BODIPY derivatives.

**Figure 4:**
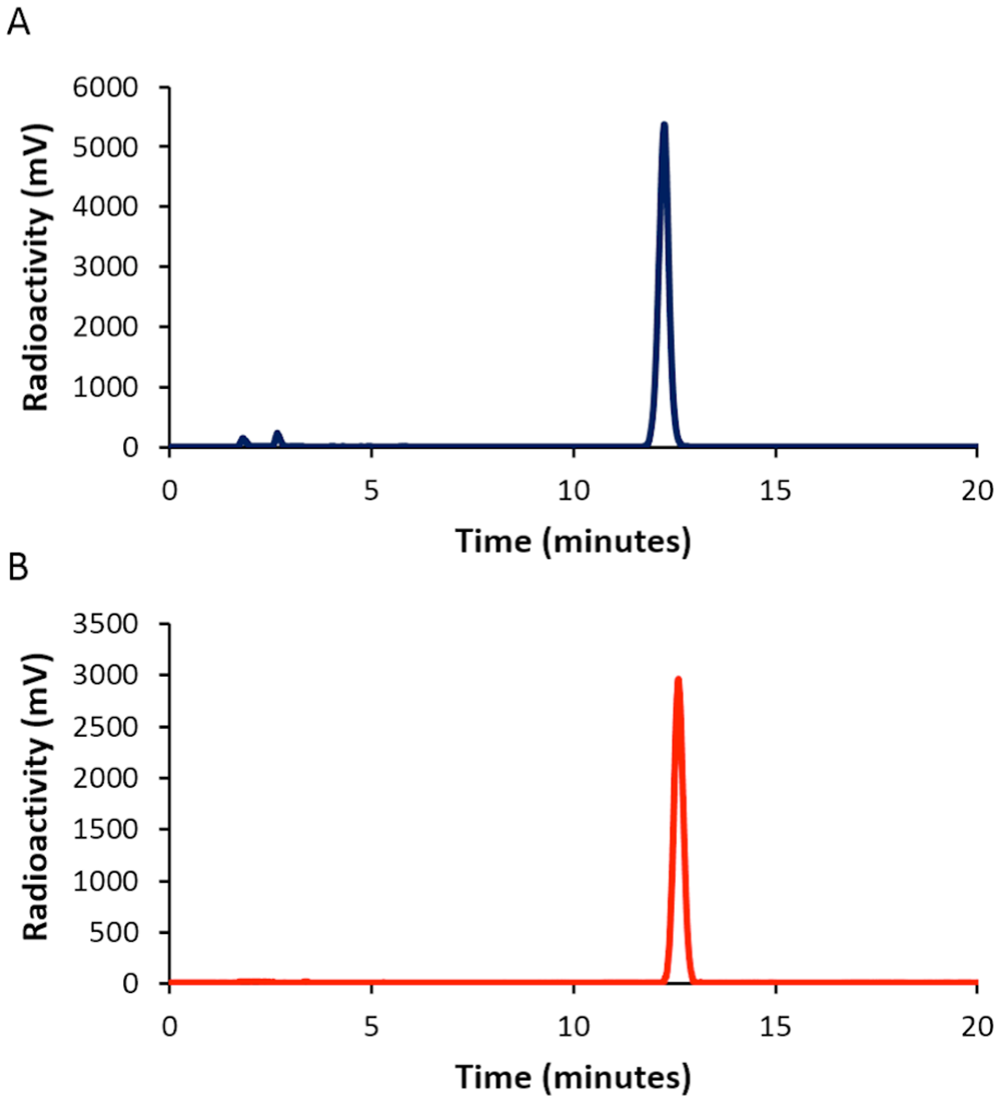
HPLC profiles of [^125^I]**2** before (**A**) and after (**B**) incubation for 24 h.

Next, in order to determine the *in vitro* stability of [^125^I]**2**, we incubated [^125^I]**2** in murine plasma for 24 h at 37 °C. When we analyzed it with HPLC after 24-h incubation of [^125^I]**2** in murine, the radioactivity peak after 24-h incubation had not changed markedly in comparison with that before incubation (Fig. 4). This result suggests that [^125^I]**2** shows high stability in murine plasma, indicating that the *in vivo* biodistribution of [^125^I]**2** should be further evaluated.

**Figure 5:**
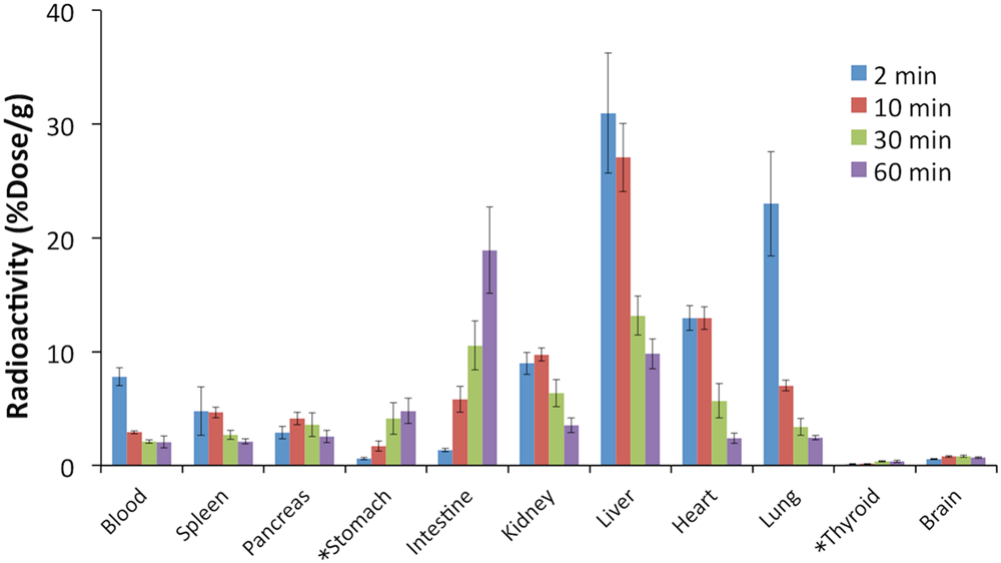
Biodistribution of radioactivity after injection of [^125^I]**2** into mice (*expressed as %Dose).

Then, we evaluated the biodistribution of radioactivity after the intravenous injection of [^125^I]**2** into normal mice (Fig. 5). The radioactivity of [^125^I]**2** after injection into mice displayed a typical biodistribution pattern for low-molecular-weight lipophilic compounds. In other words, as [^125^I]**2** was cleared from the blood, approximately 30%ID/g of [^125^I]**2** accumulated in the liver at 2 min postinjection. High radioactivity accumulation was also observed in the heart (12%ID/g) and lungs (25%ID/g) at 2 min postinjection. Thereafter, the radioactivity observed in the liver, heart, and lungs was gradually excreted into the intestine, and the radioactivity in the intestine reached 20%ID/g at 60 min postinjection. No marked radioactivity accumulation in the thyroid or stomach was observed, suggesting that [^125^I]**2** showed high stability against the deiodination reaction *in vivo* in addition to high *in vitro* stability in plasma. This also suggests that [^125^I]**2** may provide stable *in vivo* nuclear and optical dual functional probes when applied for the labeling of various proteins and peptides.

### Synthesis of ^125^I-labeled BODIPY with an active ester ([^125^I]4) and conjugation reaction of [^125^I]4 with protein and peptide

**Figure 6:**
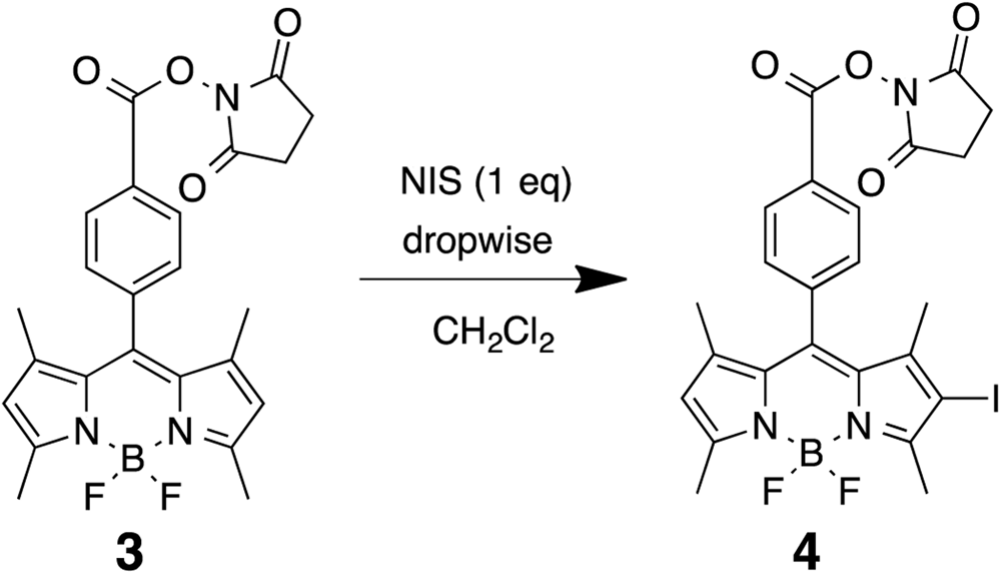
Iodination reaction of **3** (BODIPY-NHS).

**Figure 7:**
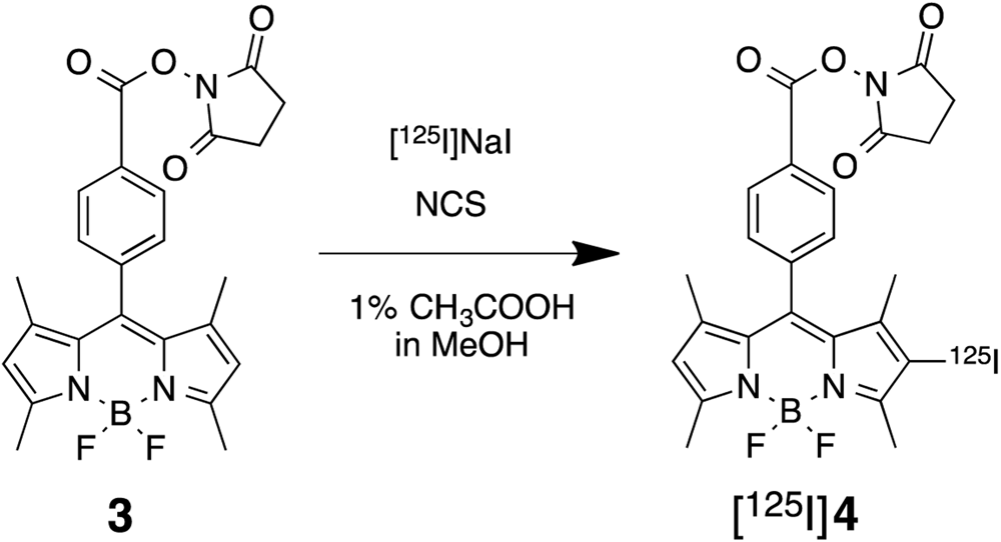
Radioiodination reaction of **3** (BODIPY-NHS).

**Figure 8:**
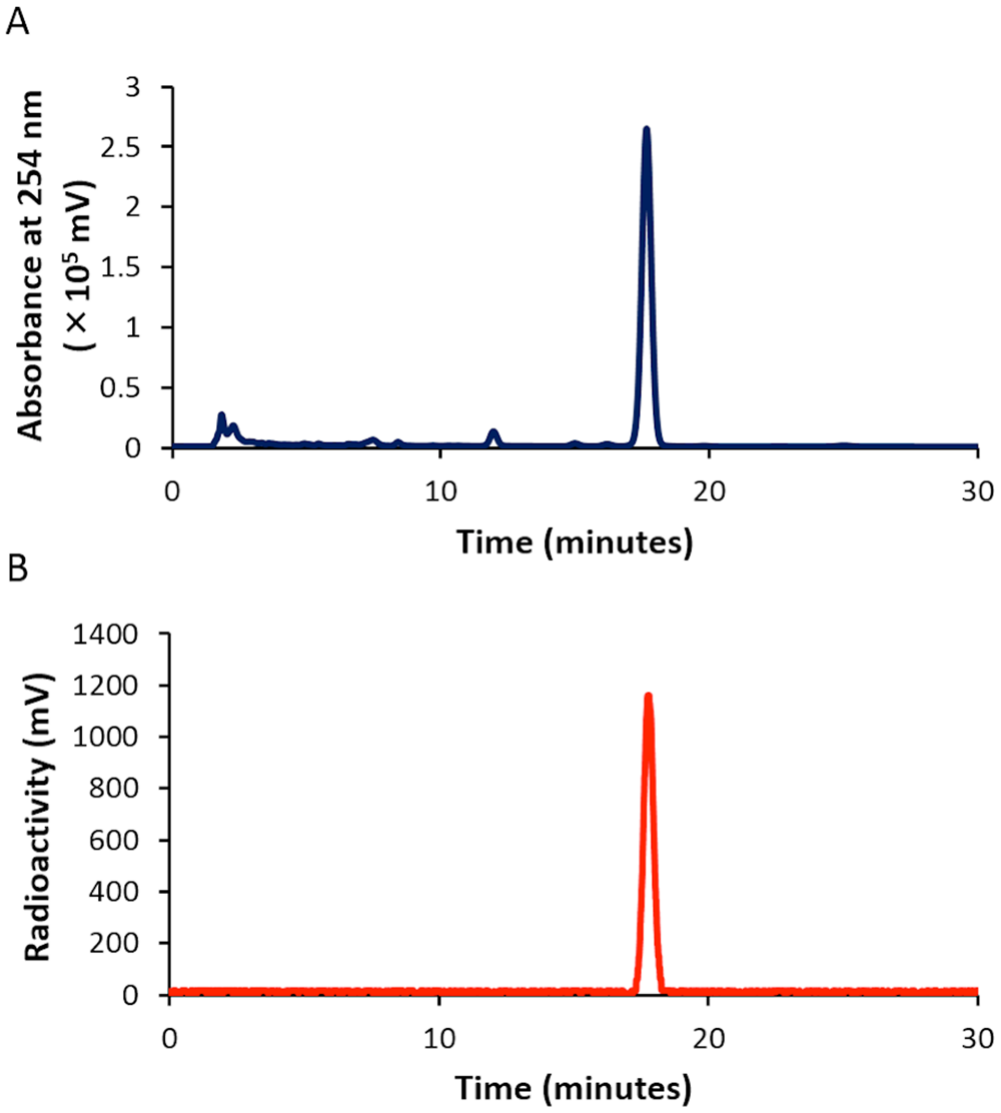
HPLC profiles of **4** (absorbance at 254 nm, **A**) and [^125^I]**4** (radioactivity, B).

Next, we designed and synthesized [^125^I]**4** with a *N*-hydroxysuccinimide (NHS) ester as a conjugation site with proteins and peptides. The nonradioactive **4** was synthesized from known compound **3** using *N*-iodosuccinimide (NIS) at a yield of 54.1% (Fig. 6). Compound **4** had maximum excitation and emission wavelengths of 523 and 541 nm, respectively, and the extinction coefficient (M^−1^cm^−1^) and quantum yield (%) of **4** were 90,600 and 11.0, respectively. Furthermore, [^125^I]**4** was synthesized by the radioiodination of compound **3** with [^125^I]NaI in the presence of *N*-chlorosuccinimde (NCS) and 10% acetic acid in methanol (Fig. 7). After purification with HPLC by the co-injection of nonradioactive compound **3**, [^125^I]**4** was successfully obtained at a radiochemical yield of 47.8% with a radiochemical purity of >99% (Fig. 8). We also attempted radioiodination with a different method using hydrogen peroxide as the oxidant, similarly to the method used in the radioiodination of [^125^I]**2**. However, we could not obtain [^125^I]**4** at high yields in this reaction, probably because the hydrolysis of the NHS ester of compound **3** occurred due to a reaction with water. Therefore, we changed to the condition of the radioiodination reaction to avoid using water as a solvent. Although we used methanol as a solvent in this reaction, no marked methyl esterification was observed.

**Figure 9:**
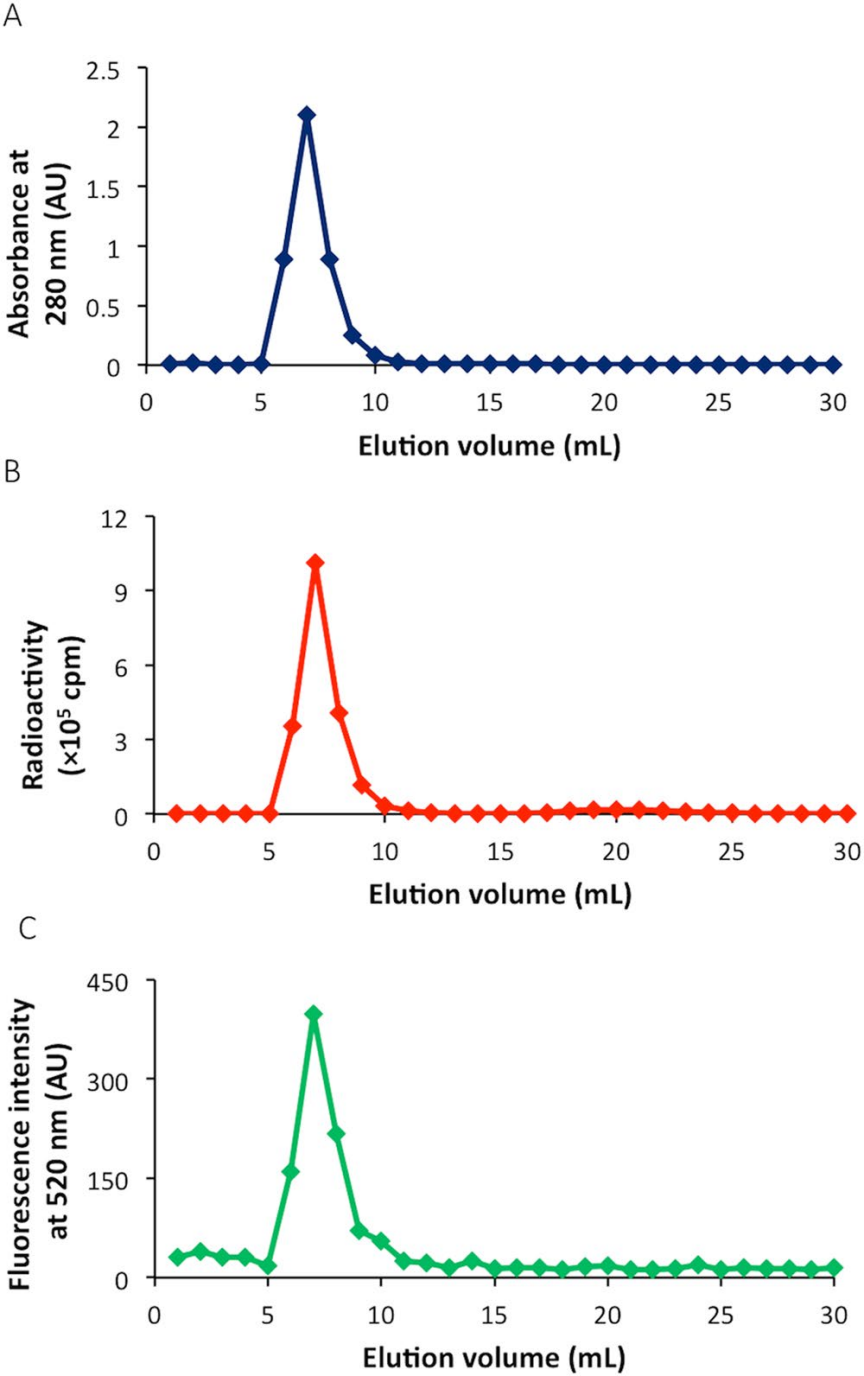
Size-exclusion chromatography of [^125^I]**4**-BSA by the detection of absorbance (280 nm) (**A**), radioactivity (**B**), and fluorescence (520 nm) (**C**).

**Figure 10:**
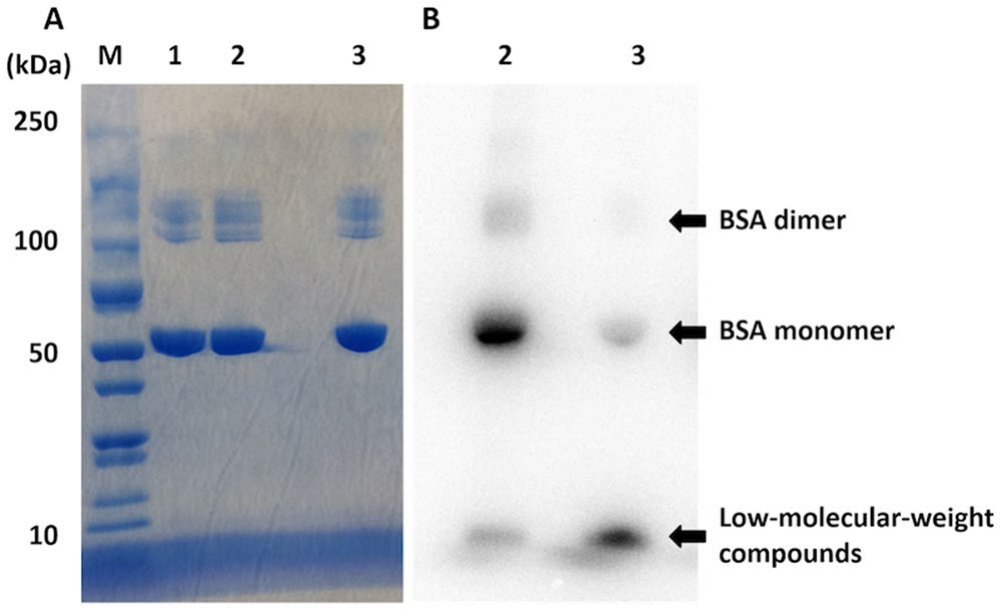
Analysis of [^125^I]**4**-BSA by SDS-PAGE. Left and right panels show CBB staining (**A**) and autoradiography (**B**), respectively. BSA (8 ng) only (lane 1, CBB staining), [^125^I]**4**-BSA (8 ng) (lane 2, CBB staining and autoradiography), and the reaction mixture of [^125^I]**2** and BSA (8 ng) (lane 3, CBB staining and autoradiography) were subjected to SDS-PAGE, respectively. Lane M shows molecular weight markers. Images were cropped to remove extraneous areas.

Next, we used [^125^I]**4** for a conjugation reaction with proteins and peptides. In the present study, we firstly selected BSA and RGD peptide as a model protein and peptide, respectively, for dual labeling with [^125^I]**4**. After the conjugation reaction of [^125^I]**4** with BSA at room temperature for 80 min, we purified [^125^I]**4**-BSA with size-exclusion chromatography. The radioactivity peak derived from [^125^I]**4**-BSA corresponded to that of absorbance at 280 nm, indicating that the conjugation of [^125^I]**4** with BSA should be successfully achieved (Fig. 9). Furthermore, when we determined the fluorescence at an emission of 520 nm, the fluorescent peak was detected at a similar elution volume to that of absorbance and radioactivity (Fig. 9). To determine whether [^125^I]**4** binds to BSA via the covalent bonds of the NHS ester of [^125^I]**4**, we analyzed [^125^I]**4**-BSA by sodium dodecyl sulfate-polyacrylamide gel electrophoresis (SDS-PAGE) (Fig. 10). The reaction mixture of [^125^I]**2** and BSA was also analyzed by SDS-PAGE as a control experiment (lane 3 in Fig. 10). As a result, most [^125^I]**4**-BSA was detected as a monomer of BSA together with a small amount of BSA dimer, and no marked radioactivity of [^125^I]**4**-BSA was found in a region where low-molecular-weight compounds were observed (Fig. 10). This means that the bonds between [^125^I]**4** and BSA should be mainly formed by the covalent bonding of the NHS ester of [^125^I]**4** with the amino groups of BSA.

**Figure 11:**
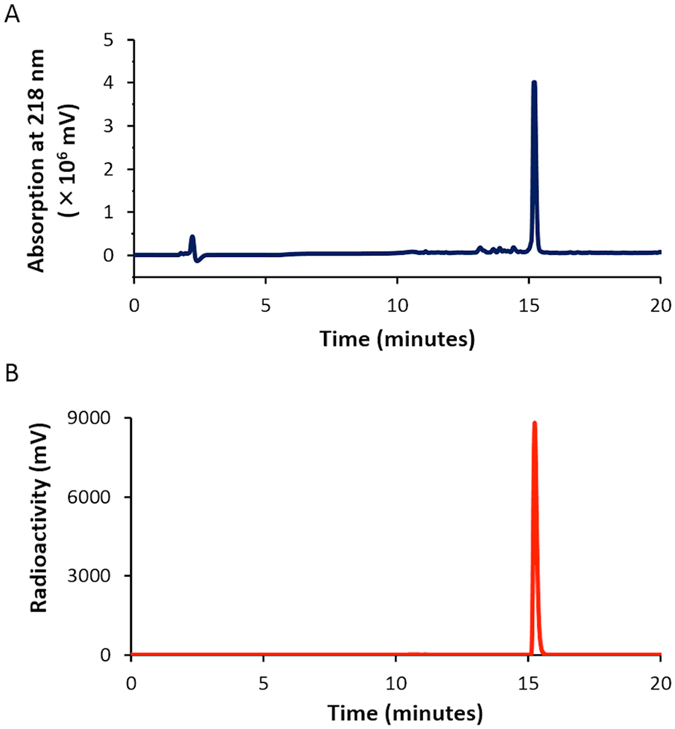
HPLC profiles of **4**-RGD (absorbance at 218 nm, **A**) and [^125^I]**4**-RGD (radioactivity, **B**).

Next, we carried out the conjugation reaction of [^125^I]**4** with RGD peptide. Many radiolabeled and fluorescent imaging probes based on RGD peptide have been studied for the *in vivo* imaging of tumors^21^. Therefore, we selected the RGD peptide as a model peptide in this study to show the basic principle for dual labeling with [^125^I]**4**. We synthesized **4**-RGD and used it as a nonradioactive standard in the HPLC analysis. The retention time of the radioactivity peak was identical to that of the absorbance of **4**-RGD, suggesting that [^125^I]**4** was successfully conjugated to RGD peptide similarly to the conjugation reaction of [^125^I]**2** with BSA (Fig. 11). The results for the conjugation reaction of [^125^I]**4** with BSA and RGD peptide also support the feasibility of nuclear and optical dual functional labeling of various other biologically active proteins and peptides including antibodies, antibody fragments, and other carriers including liposomes, micelles, and polymer conjugates^22, 23^.

**Figure 12:**
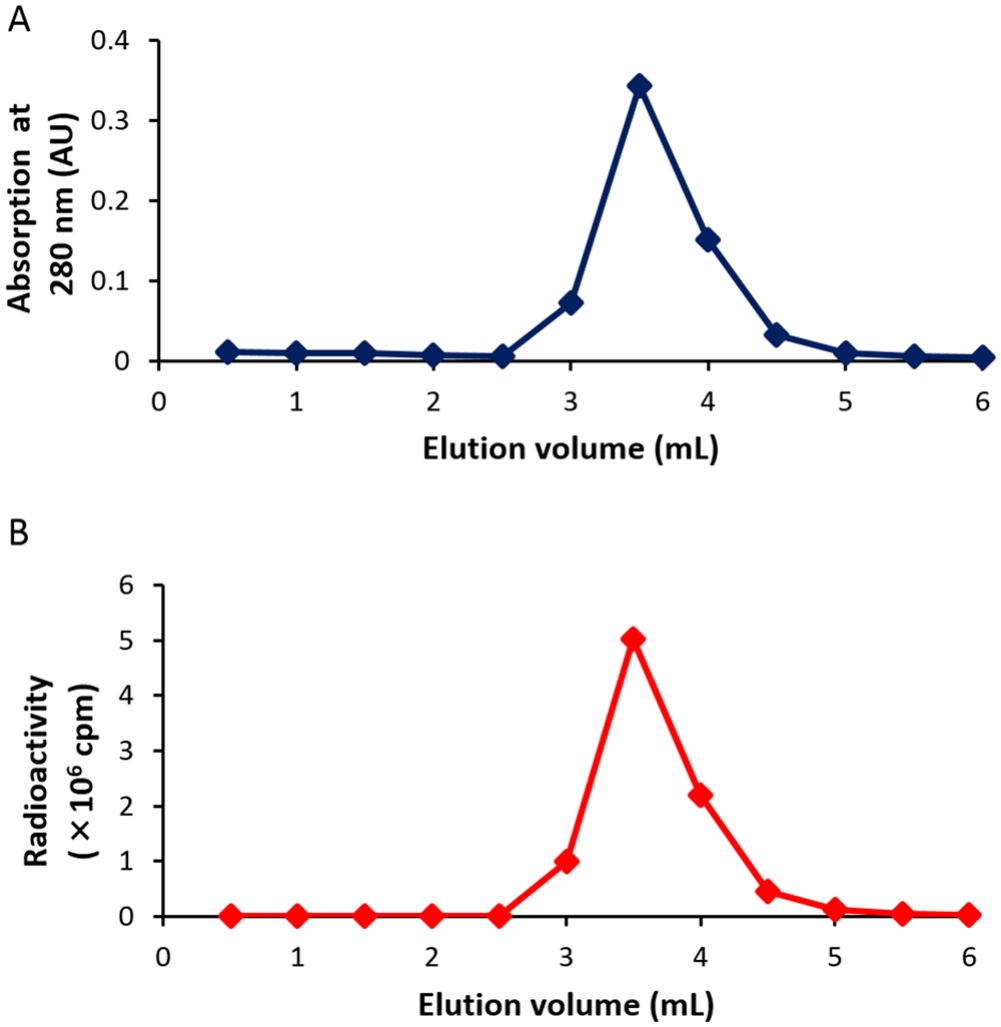
Size-exclusion chromatography of [^125^I]**4**-trastuzumab by the detection of absorbance (280 nm) (**A**) and radioactivity (**B**).

**Figure 13:**
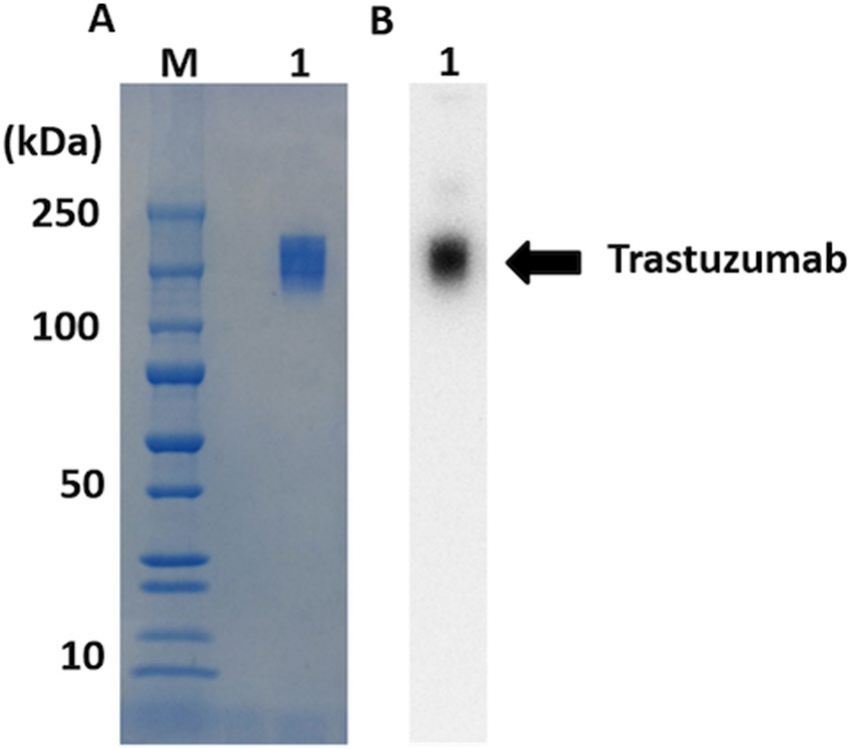
Analysis of [^125^I]**4**-trastuzumab by SDS-PAGE. Left and right panels show CBB staining (**A**) and autoradiography (**B**), respectively. [^125^I]**4**-trastuzumab (2.5 ng) was subjected to SDS-PAGE (Lane 1). Lane M shows molecular weight markers. Images were cropped to remove extraneous areas.

Then, we applied this conjugation reaction to the labeling of trastuzumab with [^125^I]**4** as one of the examples of biologically active proteins and peptides. Trastuzumab is an antibody against human epidermal growth factor receptor 2 (HER2) that is overexpressed in several cancers, and commonly used as a carrier protein of radioisotopes and fluorescence dyes for the *in vivo* imaging of cancers^24, 25^. According to the similar method used for the conjugation reaction of BSA, we reacted [^125^I]**4** with trastuzumab, and purified the reaction mixture with size-exclusion chromatography. As shown in Fig. 12, the elution volume where the radioactivity peak was detected corresponded to that of UV absorption where intact trastuzumab was eluted, indicating that trastuzumab was labeled with [^125^I]**4**. We also analyzed [^125^I]**4**-trastuzumab by SDS-PAGE. The radioactivity of [^125^I]**4**-trastuzumab was detected in a region where the intact trastuzumab was detected while no marked radioactivity was found in a region where low-molecular-weight compounds were detected (Fig. 13). This suggests that the NHS ester of [^125^I]**4** may bind to the amino groups of trastuzumab via covalent bonds, similarly to [^125^I]**4**-BSA.

**Figure 14:**
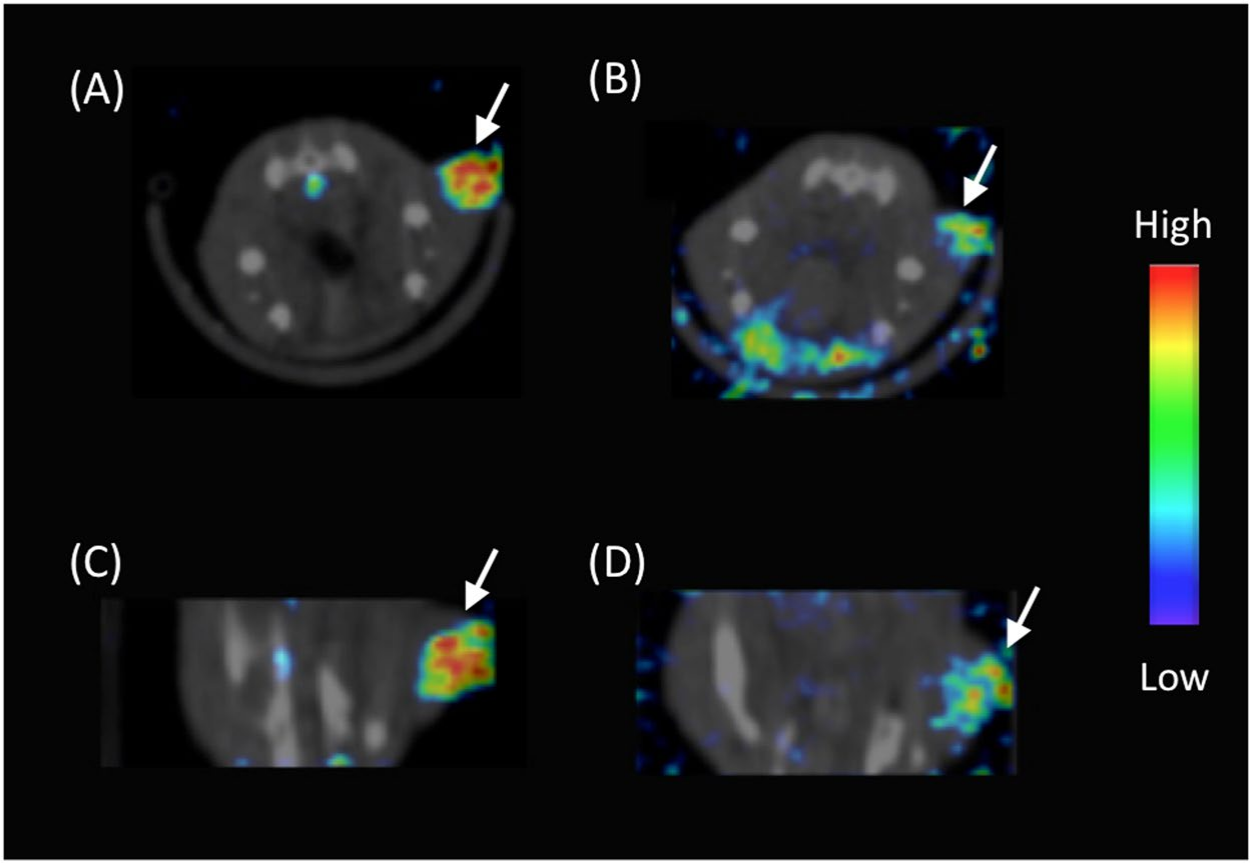
SPECT/CT images of an N87 tumor-bearing mouse at 8 h (**A** and **C**) and 24 h (**B** and **D**) after the injection of [^123^I] **4**-trastuzumab (**A** and **B**: transversal image, **C** and **D**: coronal image). The white arrows indicate the tumor.

Next, we prepared [^123^I]**4**-trastuzumab and conducted SPECT imaging studies in HER2-positive N87 tumor-bearing mice. As a result, tumors in the mice were clearly detected by SPECT imaging after the injection of [^123^I]**4**-trastuzumab (Figs. 14 and S1), suggesting that^123^ **4** may function as a radiolabeling agent without any loss of biological activity of an antibody. Since no marked radioactivity accumulation was observed in the thyroid (Fig. S1B), [^123^I]**4** shows high stability against *in vivo* deiodination reactions even after conjugation with an antibody, suggesting that radioiodine may stably bind to the BODIPY scaffold *in vivo*. In the present study, we did not perform *in vivo* fluorescence imaging due to the relatively low excitation and emission wavelengths of BODIPY **4** that are inadequate for *in vivo* fluorescent imaging. However, the successful SPECT imaging with [^123^I]**4**-trastuzumab suggests the feasibility of RI/fluorescence dual imaging, in principle, by replacing it with the new BODIPY derivative with longer excitation and emission wavelengths effective for *in vivo* imaging.

## Conclusions

In this study, for the first time, we revealed that radioiodinated BODIPY can function as a new agent useful for the nuclear and optical dual functional labeling of various biologically active proteins and peptides. Since iodine has several radioisotopes with various features suitable for use in fields from basic research to clinical application, proteins and peptides labeled with radioiodinated BODIPY may be applied extensively for not only clinical diagnosis by SPECT/fluorescence dual imaging with [^123^I]BODIPY conjugates but also radiotherapy with [^131^I]BODIPY conjugates.

## Experimental

### General

All reagents in this study were commercial products used without further purification. Sodium [^125^I]iodide ([^125^I]NaI) and [^123^I]NaI were purchased from PerkinElmer and FUJIFILM RI Pharma Co., Ltd., respectively. Smart Flash EPCLC W-Prep 2XY (Yamazen Corporation) was used for silica gel chromatography. ^1^H NMR was recorded on a JNM-ECS400 (JEOL) with tetramethylsilane (TMS) as an internal standard. Coupling constants are reported in Hertz (Hz). Multiplicity was defined as singlet (s), doublet (d), triplet (t), multiplet (m), and quartet (q). ESI mass spectrometry was conducted with a Shimadzu LCMS-2020. High-resolution mass spectrometry (HRMS) was conducted with JMS-700V (JEOL). High-performance liquid chromatography (HPLC) was performed with Shimadzu system (an LC-20AD pump with an SPD-20A UV detector, λ = 254 nm) using a Cosmosil C_18_ column (Nacalai Tesque, COSMOSIL 5C_18_-AR-II 4.6 mm I.D. × 150 mm) and CH_3_CN/H_2_O (60: 40) as the mobile phase at a flow rate of 1.0 mL/min. HER2-expressing human gastric cancer cells (N87) were purchased from DS Pharma Biomedical (Osaka, Japan).

### Chemistry

Compounds **1**, **2**, and **3** were synthesized according to the previous procedures reported by Wang *et al*.^26^, Chen *et al*.^17^, and Nepomnyashchii *et al*.^27^, respectively.

### 2-Iodo-8-(4-(*N*-succinimidoxycarbonyl)phenyl)−1,3,7,9-tetramethyl-BODIPY (4)

To a solution of BODIPY derivative **3** (100.0 mg, 0.21 mmol) in CH_2_Cl_2_ (25 mL), *N*-iodosuccinimide (NIS) (48.4 mg, 0.21 mmol) in CH_2_Cl_2_ (10 mL) was added dropwisely and the reaction mixture was stirred at 0 °C for 30 min. After the addition, the reaction mixture was stirred at room temperature for 1 h. The reaction mixture was evaporated under a vacuum. The residue was purified by silica gel chromatography (CHCl_3_/n-hexane = 10/1) to provide 68.7 mg of **4** at a yield of 54.1%. ^1^H NMR (400 MHz, (CDCl_3_) δ 8.30 (d, *J = *8.40 Hz, 2 H), 7.49 (d, *J* = 8.40 Hz, 2 H), 6.08 (s, 1 H), 2.96 (s, 4 H), 2.64 (s, 3 H), 2.58 (s, 3 H), 1.38 (s, 6 H); ^13^C NMR (100 MHz, CDCl_3_) δ 14.8, 15.0, 17.1, 25.6, 84.8, 122.7, 126.0, 128.9, 130.1, 131.1, 131.4, 138.9, 141.8, 143.0, 144.7, 155.4, 158.6, 161.1, 169.1. HRMS (FAB) 591.0625 [M^+^] (Chemical formula: C_24_H_21_BF_2_IN_3_O_4_, calculated m/z value: 591.0638).

### RGD peptide conjugated with 4 (4-RGD)

**4**-RGD was synthesized according to a method reported previously^15^. BODIPY derivative **4** (0.89 mg, 1.5 µmol) and c(RGDyK) (0.40 mg, 0.65 µmol, used as RGD) in DMSO (50 µL) were mixed and *N,N*-diisopropylethylamine (5 µL) was added to the mixture. After 3-h incubation, the reaction mixture was purified by reversed-phase HPLC. The flow rate was 1 mL/min, with the mobile phase starting from 90% solvent A (0.1% TFA in water) and 10% solvent B (0.1%TFA in CH_3_CN) to 0% solvent A and 100% solvent B at 20 min. ^1^H NMR (400 MHz, CD_3_OD) δ 8.05 (d, *J* = 8.40 Hz, 2 H), 7.50 (d, *J* = 8.00 Hz, 2 H), 7.00 (d, *J = *8.40 Hz, 2 H), 6.69 (d, *J* *=* 8.80 Hz, 2 H), 6.19 (s, 1 H), 4.65–4.60 (m, 1 H), 4.46–4.44 (m, 1 H), 4.32–4.26 (m, 2 H), 3.95–3.91 (m, 1 H), 3.49–3.45 (m, 2 H), 3.13–3.12 (m, 1 H), 2.89–2.80 (m, 3 H), 2.62–2.52 (m, 8 H), 1.60–1.49 (m, 7 H), 1.43–1.42 (m, 7 H), 1.34–1.29 (m, 7 H), 1.11–1.06 (m, 1 H), 0.91–0.88 (m, 2 H). MS (ESI) m/z 1096.7 [M + H^+^] (Chemical formula: C_47_H_58_BF_2_IN_11_O_9_, calculated m/z value: 1096.3).

### Determination of fluorescence parameters

The fluorescence parameters including fluorescence excitation/emission wavelength and intensity were measured by an RF-5300 and RF-1500 fluorescence spectrophotometer (Shimadzu).

### Preparation of [^125^I]2

To a solution of **1** (1 mg/mL, 50 µL EtOH), [^125^I]NaI (1.3–1.7 MBq), 1 N HCl (50 µL), and 3% H_2_O_2_ (50 µL) were added. The reaction was allowed to proceed at room temperature for 60 min and terminated by the addition of saturated NaHSO_3_ aq. (100 µL). The reaction mixture was neutralized by adding a solution of saturated NaHCO_3_ aq. (200 µL) and extracted with ethyl acetate. After the organic phase was dried by passing through a column filled with anhydrous Na_2_SO_4_, the solution was dried with a stream of argon gas. [^125^I]**2** was purified by the reversed-phase HPLC on a COSMOSIL 5C_18_-AR-II column with an isocratic solvent of CH_3_CN/H_2_O = 4/1 at a flow rate of 1 mL/min.

### Preparation of [^123/125^I]4

BODIPY derivative **3** was dissolved in methanol containing 1% acetic acid (0.5 mg/mL, 50 µL). To this solution, *N*-chlorosuccinimide (NCS) in methanol (0.5 mg/mL, 20 µL) and [^123/125^I]NaI (1.5–3.8 MBq or 111 MBq for ^125^I or ^123^I labeling, respectively) were added. After the reaction mixture was incubated at room temperature for 40 min, saturated NaHSO_3_ aq. (100 µL) was added to quench the reaction. The reaction mixture was neutralized by adding a solution of saturated NaHCO_3_ aq. (200 µL) and extracted with ethyl acetate. After the organic phase was dried by passing through a column filled with anhydrous Na_2_SO_4_, the solution was dried with a stream of argon gas. [^123/125^I]**4** was purified by the reversed-phase HPLC on a COSMOSIL 5C_18_-AR-II column with an isocratic solvent of CH_3_CN/H_2_O = 3/2 at a flow rate of 1 mL/min.

### Conjugation of [^125^I]4 with BSA

To a solution of bovine serum albumin (BSA) in 50 mM borate buffer (pH 8.5) (1.88 mg/mL, 1.0 mL), [^125^I]**4** (185 kBq, 50 µL) was added. The reaction mixture was incubated at room temperature for 80 min. Thereafter, a solution of the reaction mixture was fractionated every 0.5 mL by size-exclusion chromatography with the PD-10 column eluted with 5% DMF in 50 mM borate buffer (pH 8.5). The radioactivity in each fraction was measured, and the fractions that corresponded with the absorbance of BSA were calculated as [^125^I]**4**-BSA.

### Conjugation of [^125^I]4 with RGD peptide

We prepared RGD peptide-conjugated [^125^I]**4** on the basis of a method reported previously^15^. To a mixture of [^125^I]**4** (0.66 MBq) and c(RGDyK) (0.2 mg) in DMSO (50 µL) was added DIPEA (2 µL). After 2-h incubation, the reaction mixture was purified with HPLC to yield [^125^I]**4**-RGD at an 82.0% radiochemical yield and over 99% radiochemical purity.

### Conjugation of [^123/125^I]4 with trastuzumab

We added [^123/125^I]**4** (185 kBq or 24–30 MBq for ^125^I or ^123^I labeling, respectively) in DMSO (50 µL) and PBS (pH 8.6, 500 μL) to a solution of trastuzumab in PBS (pH 8.6, 1 mg/mL, 500 μL). The reaction mixture was incubated at room temperature for 80 min. Thereafter, a solution of the reaction mixture was fractionated every 0.5 mL by size-exclusion chromatography with the PD-10 column eluted with PBS (pH 8.6). The radioactivity in each fraction was measured, and the fractions that corresponded with the absorbance of trastuzumab were calculated as [^125^I]**4**-trastuzumab.

### SDS PAGE analysis of [^125^I]4-BSA and [^125^I]4-trastuzumab

We analyzed [^125^I]**4**-BSA and [^125^I]**4**-trastuzumab with SDS-PAGE with a 10−20 and 5–20% gradient polyacrylamide gel (ATTO) for [^125^I]**4**-BSA and [^125^I]**4**-trastuzumab, respectively. A protein molecular weight marker was used simultaneously. After electrophoresis at 20 mA for 60 and 65 min for [^125^I]**4**-BSA and [^125^I]**4**-trastuzumab, respectively, the gel was stained with Coomassie Brilliant Blue (CBB) G-250 (Invitrogen, Simple Blue SafeStain) to determine the molecular weight of [^125^I]**4**-BSA and [^125^I]**4**-trastuzumab. Furthermore, the gel was exposed to a BAS imaging plate (Fuji Film). Autoradiographic images were obtained using a BAS 5000 scanner system (Fuji film).

### *In vitro* stability of [^125^I]2 in plasma

[^125^I]**2** (50 µL in a mixed solvent of 90% saline and 10% EtOH, 0.11–0.22 MBq) was diluted with freshly prepared murine plasma (250 µL). The solution was incubated at 37 °C for 24 h. After the addition of acetonitrile (600 µL) and vortexing, the mixture was centrifuged (4 °C, 10,000 *g*, 5 min). The supernatant was filtered and the filtrate was analyzed by reversed-phase HPLC using a COSMOSIL column (5C_18_ AR-II) with an isocratic solvent of CH_3_CN/H_2_O (4/1) at a flow rate of 1.0 mL/min.

### *In vivo* biodistribution in normal mice

Animal experiments were conducted in accordance with our institutional guidelines and approved by Kyoto University. A saline solution (100 μL) containing 0.1% tween 80 and 10% ethanol of [^125^I]**2** (37 kBq) was injected intravenously into the tails of ddY mice (5 weeks old, male, 5 animals per each group). The mice were sacrificed at 2, 10, 30, and 60 min postinjection. The organs of interest were removed and weighed, and the radioactivity was measured with an automatic γ counter (Wizard 1470, PerkinElmer). The results were calculated as the percentage injected dose per gram (%Dose/g) or percentage injected dose (%Dose). Values are expressed as the mean ± SD.

### SPECT/CT study

N87 (human gastric cancer cell) tumor-bearing mice were prepared as reported previously^28^. [^123^I]**4**-trastuzumab (2.2–4.1 MBq/ 60–150 μg, 150 μL PBS) was intravenously injected into the N87 tumor-bearing mice (n = 3). The mice were anesthetized by isoflurane (2–2.5%), and SPECT and CT images were obtained using the U-SPECT-II/CT system (MILabs, Utrecht, the Netherlands) with 0.6-mm pinhole collimators (SPECT conditions: 60 min × 1 frame; CT conditions: accurate full angle mode in 65 kV/615 μA) at 8 and 24 h after injection of [^123^I]**4**-trastuzumab. SPECT images were reconstructed using the OSEM method (8 subset, 1 iteration) with a 0.6-mm Gaussian filter.

## Electronic supplementary material

supplementary info
