# Supplementary material for: Radioiodination of BODIPY and its application to a nuclear and optical dual functional labeling agent for proteins and peptides
Source: Sci Rep. 2017 Jun 13;7:3337. doi: 10.1038/s41598-017-03419-z (PMC5469783; doi:10.1038/s41598-017-03419-z)

**Supporting Information for**

**Radioiodination of BODIPY and Its Application to a Nuclear and Optical Dual Functional Labeling Agent for Proteins and Peptides**

Masahiro Ono^1^,^*^, Hiroyuki Watanabe^1^, Yuki Ikehata^1^, Ning Ding^1^, Masashi Yoshimura^1^, Kohei Sano^1^, Hideo Saji^1^

^1^Department of Patho-Functional Bioanalysis, Graduate School of Pharmaceutical Sciences, Kyoto University

*To whom correspondence should be addressed. Phone: +81-75-753-4608, Fax: +81-75-753-4568, e-mail: [ono@pharm.kyoto-u.ac.jp](mailto:ono@pharm.kyoto-u.ac.jp) for M. Ono.

**SPECT/CT study**

A N87 (human gastric cancer cell) tumor-bearing mouse was prepared as reported previously. [^123^I]**4**-trastuzumab (4.1 MBq/150 μg, 150 μL PBS) was intravenously injected into the N87 tumor-bearing mouse. The mouse were anesthetized by 2.5% isoflurane, and SPECT and CT images were obtained using the U-SPECT-II/CT system (MILabs, Utrecht, the Netherlands) with 1.0-mm pinhole collimators (SPECT conditions: 60 min × 1 frame; CT conditions: accurate full angle mode in 65 kV/615 μA) at 25 h after injection of [^123^I]**4**-Trastuzumab. SPECT images were reconstructed using the OSEM method (8 subset, 1 iteration) with a 1.0-mm Gaussian filter.


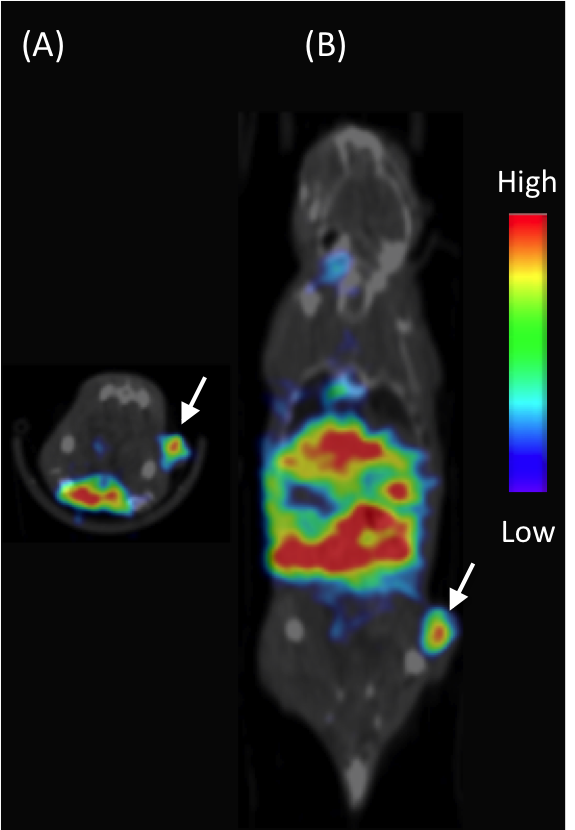


Figure S1. SPECT/CT images of N87 tumor-nearing mouse at 25 h after injection of [^125^I]**4**-Trastuzumab (A: transversal image, B: coronal image). The white arrows indicate the tumor.

**NMR spectra of BODIPY 4**


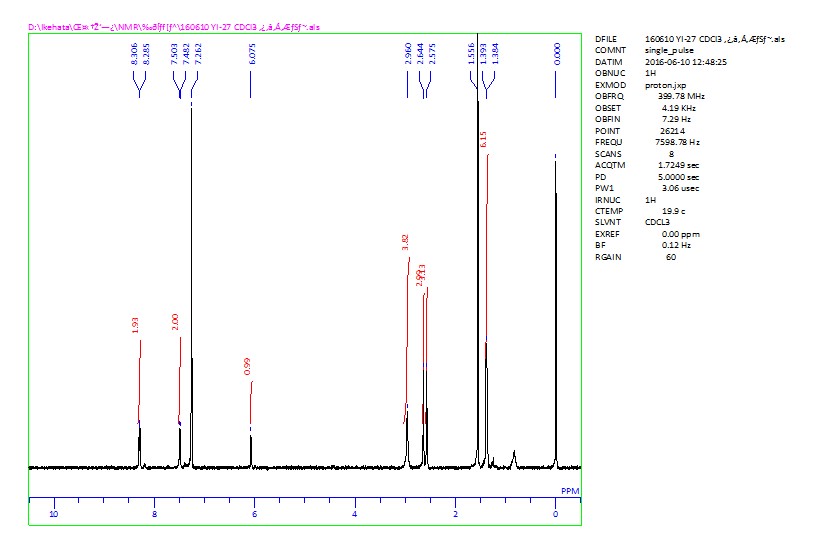


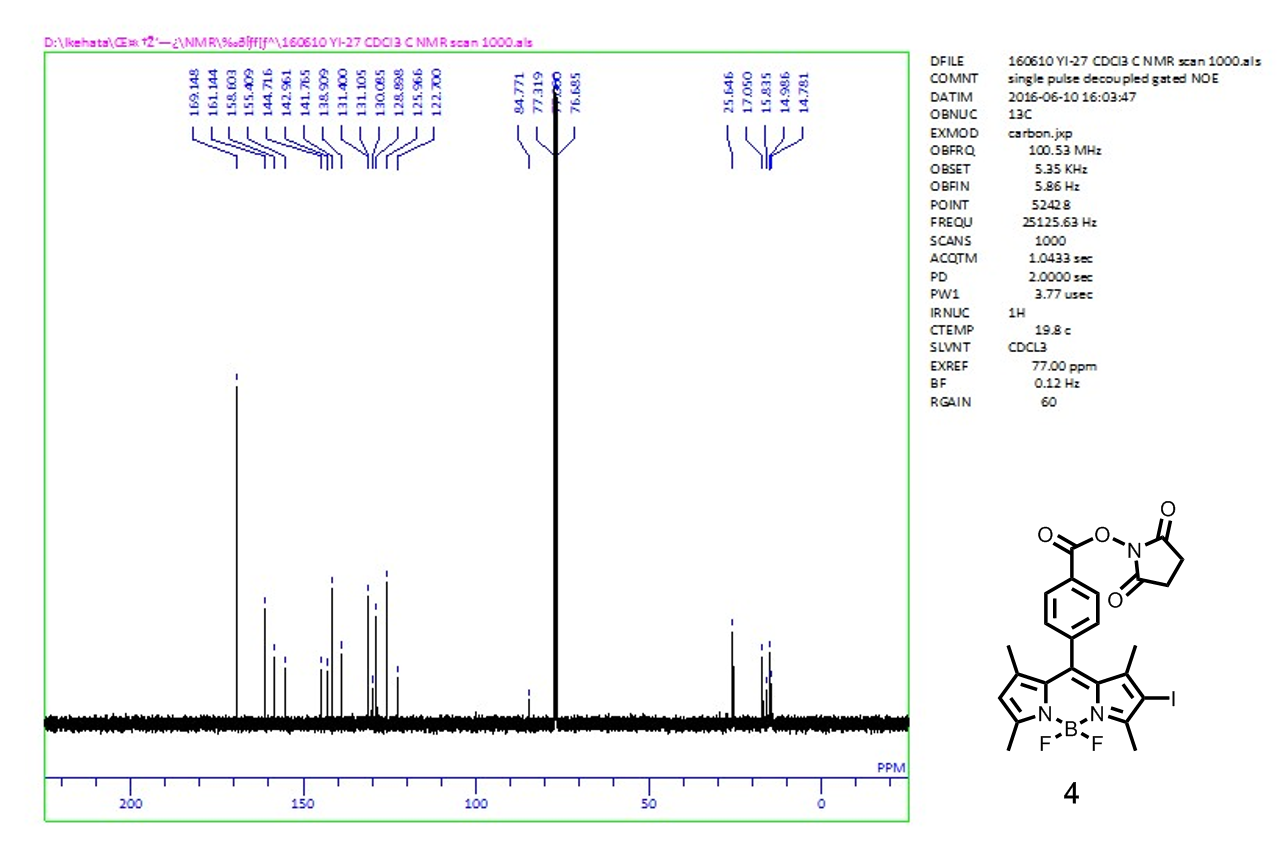


**MS spectrum of 4-RGD**


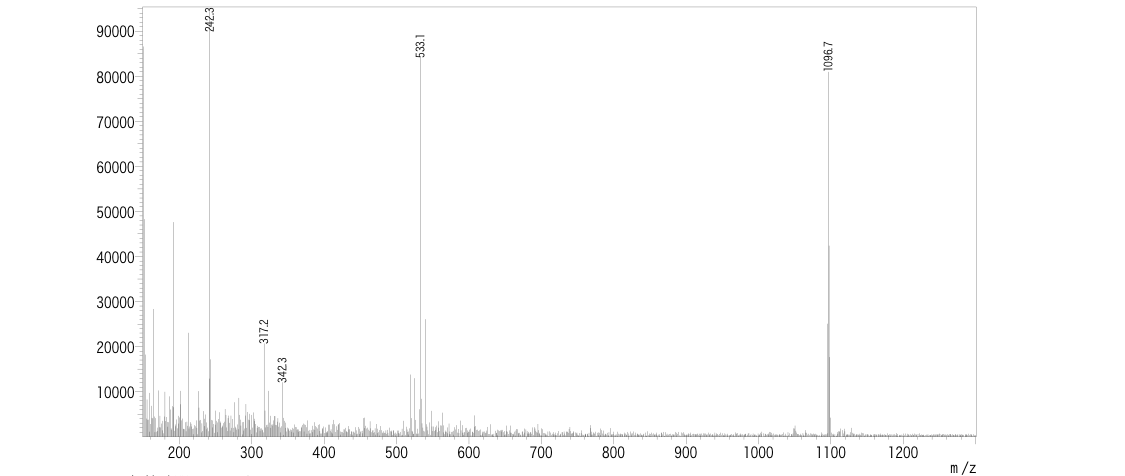

Supplement: Supplementary file 1 — supplementary info [file 41598_2017_3419_MOESM1_ESM.docx]
